# Lactational Stage of Pasteurized Human Donor Milk Contributes to Nutrient Limitations for Infants

**DOI:** 10.3390/nu9030302

**Published:** 2017-03-18

**Authors:** Christina J. Valentine, Georgia Morrow, Amanda Reisinger, Kelly A. Dingess, Ardythe L. Morrow, Lynette K. Rogers

**Affiliations:** 1 The Division of Obstetrics and Gynecology, The University of Cincinnati, Cincinnati, OH 45220, USA; 2 OhioHealth Mothers’ Milk Bank of Ohio, Columbus, OH 43215, USA; georgiamor@gmail.com; 3 Akron Children’s Hospital, Akron, OH 44308, USA; ahodge@gmail.com; 4 Biomolecular Mass Spectrometry and Proteomics, Utrecht Institute for Pharmaceutical Sciences, University of Utrecht, Padualaan 8, 3584 CD Utrecht, The Netherlands; k.a.dingess@uu.nl; 5 Cincinnati Children’s Hospital, Cincinnati, OH 45229, USA; Ardythe.Morrow@cchmc.org; 6 Center for Perinatal Research, The Research Institute at Nationwide Children’s Hospital, Columbus, OH 43215, USA; Lynette.Rogers@Nationwidechildrens.org

**Keywords:** preterm infants, human milk, nutrition, donor milk, DHA, lysine

## Abstract

Background. Mother’s own milk is the first choice for feeding preterm infants, but when not available, pasteurized human donor milk (PDM) is often used. Infants fed PDM have difficulties maintaining appropriate growth velocities. To assess the most basic elements of nutrition, we tested the hypotheses that fatty acid and amino acid composition of PDM is highly variable and standard pooling practices attenuate variability; however, total nutrients may be limiting without supplementation due to late lactational stage of the milk. Methods. A prospective cross-sectional sampling of milk was obtained from five donor milk banks located in Ohio, Michigan, Colorado, Texas-Ft Worth, and California. Milk samples were collected after Institutional Review Board (#07-0035) approval and informed consent. Fatty acid and amino acid contents were measured in milk from individual donors and donor pools (pooled per Human Milk Banking Association of North America guidelines). Statistical comparisons were performed using Kruskal–Wallis, Spearman’s, or Multivariate Regression analyses with center as the fixed factor and lactational stage as co-variate. Results. Ten of the fourteen fatty acids and seventeen of the nineteen amino acids analyzed differed across Banks in the individual milk samples. Pooling minimized these differences in amino acid and fatty acid contents. Concentrations of lysine and docosahexaenoic acid (DHA) were not different across Banks, but concentrations were low compared to recommended levels. Conclusions. Individual donor milk fatty acid and amino acid contents are highly variable. Standardized pooling practice reduces this variability. Lysine and DHA concentrations were consistently low across geographic regions in North America due to lactational stage of the milk, and thus not adequately addressed by pooling. Targeted supplementation is needed to optimize PDM, especially for the preterm or volume restricted infant.

## 1. Introduction

The American Academy of Pediatrics (AAP) [1] and the World Health Organization (WHO) [2] recommend mother’s own human milk feeding because of immunological benefits which lessen disease in the high-risk neonate [3,4]. Epidemiological investigations have determined that feeding mother’s own milk decreases the likelihood of necrotizing enterocolitis and late-onset sepsis in preterm infants [4,5,6], resulting in shorter hospital stays and decreased cost of care [7,8]. When mother’s own milk is not available, pasteurized donor milk (PDM) is used as a reasonable alternative for the preterm infant [9,10,11,12,13]. To achieve this practice, mother’s own milk is often augmented with pasteurized donor milk [14]. In fact, recent retrospective work demonstrated cost effectiveness in the intensive care unit that optimized human milk feeding [15]. However, a major gap in knowledge is the nutritional consequences of feeding infants with low volumes of PDM that is often provided by mothers feeding older term infants who consume much greater quantities of milk and receive food sources at 4–6 months [16]. Furthermore, whether from the mother or donor, specific human milk components can be influenced by maternal diet [17]. The reality is that many preterm infants fed even fortified human milk may demonstrate growth, meeting specified weight goals, but 43% are still documented as small for gestational age and are in the lower growth percentiles at discharge—unlike their term counterparts [18,19,20]. Most concerning is that growth failure—particularly linear velocity—is associated with neurodevelopmental morbidities [21,22,23]. In addition, a recent multi-site trial randomly assigning infants to donor milk or preterm formula did not find developmental advantages [24]. Consequently, understanding the nutrient characteristics of pooled human milk is essential to improving fortification strategies.

The composition of human milk changes throughout the course of lactation as the infant matures, and these changes are consistent with infants consuming increasing quantities of milk and ingesting other food sources [25,26]. Consequently, the nutrient composition of most PDM alone is not considered adequate to meet the needs of the preterm or volume-restricted infant due to the late lactational stage of the donor milk, which is nutritionally less concentrated than early milk [27,28], and there is consensus that PDM requires fortification [29,30,31,32]. Furthermore, a recent meta-analysis of term and preterm human milk composition confirmed that human milk is profoundly variable [33]. There are a multitude of factors that may contribute to this variability, including stage of lactation, diurnal cycles, exposure to environmental contaminants such as cigarette smoke, and the treatment of expressed milk (i.e., storage, containers, or tubing delivery) [33,34,35]. While many of these variables are normalized in mother’s own milk fed consistently around the clock and for months, milk obtained for donation is subject to the circumstances at that time. Expert opinion suggests more research is needed in the area of donor milk and fortification [11]. As a starting point to address the most basic elements in donor milk composition, we chose to measure fatty acid and amino acid contents. The purpose of this study was to determine the variability in fatty acid and amino acid composition in individual and pooled PDM using current pooling practices in place at milk banks compliant with Human Milk Banking Association of North America (HMBANA) guidelines, specifically for protein (0.7–1.0 g/100 mL) and caloric contents (67–81 kcal/100 mL) [36].

## 2. Materials and Methods

### 2.1. Study Design/Participants

This is a prospective cross-sectional sampling of human milk from five HMBANA member milk banks: California, Colorado, Texas-Ft Worth, Michigan, and Ohio. Prospective enrollees were mothers donating to milk banks between December 2008 and June 2010. Participants were enrolled after Institutional Review Board approval and informed consent of the human milk donors. Milk samples from 15 to 16 individual mothers per Bank were collected from December 2008 through November of 2010. Collected samples were pasteurized, and 1 mL was removed and immediately stored at −80 °C for laboratory measurements. The remaining milk was pooled into five pools per bank to meet the required caloric content and protein concentrations following HMBANA guidelines [37]. The samples were shipped frozen to Nationwide Children’s Hospital, Columbus, OH, USA and stored at −80 °C until analyzed.

### 2.2. Nutrient Analyses

Fatty acid concentrations were determined by gas chromatography and included capric (C10:0), lauric (C12:0), myristic (C14:0), palmitic (C16:0), palmitoleic (C16:1ω7), steric (C18:0), oleic (C18:1ω9), vaccenic (C18:1ω7), linoleic (18:2ω6), α-linolenic (18:3ω3), γ-linolenic (C18:3ω3), arachidonic (20:4ω6), eicosapentaenoic (20:5ω3), and docosahexaenoic (22:6ω6) acids, as described previously [28]. Amino acid concentrations were measured by high pressure liquid chromatography and included phosphoserine, taurine, phosphoethanolamine, aspartic acid, threonine, serine, glutamic acid, alpha-aminoadipic, proline, glycine, alanine, valine, methionine, isoleucine, leucine, tyrosine, phenylalanine, tryptophan, lysine, histidine, and arginine, as described previously [28]. Each individual donor milk sample and the pooled samples from each milk bank were analyzed independently.

### 2.3. Statistical Analysis

Statistical comparisons were performed using Kruskal–Wallis for the demographic data (Table 1). Fatty acid and amino acid contents were compared across Centers with multivariate analysis of variance using a linear regression model. Results of the multivariate tests are reported in the figure legends, and coefficient of variation (adjusted *R*^2^) and the between-subject differences (*p*-values) are reported within the graph. All milk composition data were analyzed using lactational stage as a co-variate. Correlations between lactational stage and amino acids or fatty acids were performed by Spearman’s correlation. Statistical significance was set at *p* < 0.05 for all analyses.

## 3. Results

### 3.1. Participants

Women currently donating to HMBANA member banks were included in this study. Prior to donating, the participants received rigorous medical, dietary, and laboratory screening. No women refused participation. There were 16 women enrolled from California and Texas, and 15 women enrolled from each site in Colorado, Michigan, and Ohio. Participant median ages across Centers were 31.5–33.5 years (range 20–42 years) and median lactational stages were 1–5.5 months (range 0–13 months) (Table 1).

### 3.2. Nutrient Analyses

After caloric content and protein measurements were performed in the individual samples, the milk samples at each individual site were pooled to achieve an overall caloric content of 67–81 kcal per 100 mL and an overall total protein content of 0.7–1 g per 100 mL. Protein-to-calorie ratio with these combinations reaches 3.3 with standard fortification; however, baseline assumptions may change with mother’s lactational stage.

**Figure 1:**
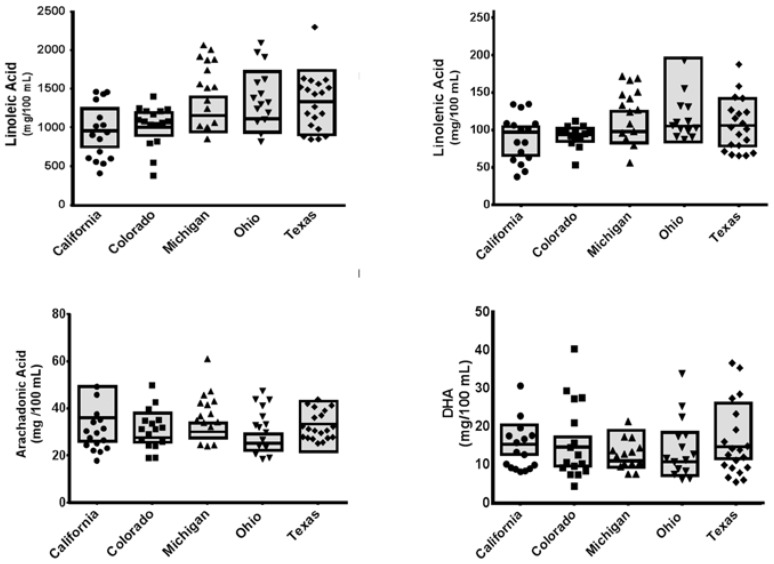
Fatty acid levels in pasteurized donor milk samples. The individual variability of linoleic acid, linolenic acid, arachidonic acid, and docosahexaenoic acid (DHA) were calculated as mg/kg/day, assuming 150 mL per day and a 1 kg infant. Individuals within each Bank are represented by the small symbols. The boxes represent the median and range for the pooled samples. Data are representative of 15–16 amino acids; analyses in individual milk samples revealed that all but two of the measured amino acids were different across Banks—these were taurine and tryptophan (Figure 2 and Table S2). Once samples were pooled, no statistical differences in amino acid contents across Banks were indicated (Table 3).

Fatty acid analysis of the individual samples revealed substantial differences across Banks in all fatty acids ranging from C16:0 to C20:5ω3 (Figure 1 and Table S1). Fatty acid analysis of the pooled samples indicated no differences between Centers (Table 2); however, the average docosahexaenoic acid (DHA) concentration in all samples was 7.1 mg/100 mL, which is substantially lower than the 65 mg/100 mL (0.8 mol %) required to match fetal accretion concentrations (based on intakes of 150 mL/kg/day) [38].

The composition of human milk is highly dependent upon lactational stage, which was highly significant in the multivariate regression analyses. Consequently, we analyzed both amino acids and fatty acids for correlation with lactational stage in individual and in pooled samples. In the individual samples, all amino acids were negatively correlated with lactational stage (*p* ≤ 0.006), but these correlations were no longer significant after the samples were pooled (*p* ≥ 0.131). Only the fatty acids eicosapentaenoic acid (EPA, 20:5ω3) (*p* = 0.029) and docosahexaenoic acid (DHA, 22:6ω3) (*p* = 0.025) were significantly correlated with lactational stage in the individual samples, and these correlations were no longer significant after the samples were pooled (*p* = 0.819 and *p* = 0.690, respectively).

## 4. Discussion

Mother’s own human milk is recommended by the AAP as a unique biologic source of nutrition for both term and preterm infants [1]. When mother’s milk is not available, PDM is a reasonable alternative for the preterm infant; however, PDM does not meet the nutritional needs of infants that are preterm or ingesting low volumes of milk if fed as the sole source of nutrition, and thus requires supplementation [9,10,11,39]. While women donating to human milk banks undergo extensive medical screening, there are many variables that are not controlled. The milk content of fatty acids of an individual woman may vary substantially depending upon her recent diet and/or other demographic factors. Human milk collection, storage, and fortification and biologic components are often described [40], but in light of the recent clinical practice of using donor milk, and publications on variability in human milk, the effectiveness of pooling to adequately address the nutritional differences in individual donor milk needs to be evaluated [33].

Our data indicate that donor milk protein-to-calorie ratio is within the range for nitrogen retention of 2.6–3.6 [41]; however, these studies were done with preterm formula, and other factors such as individual variation, heat treatment, absorption, or micronutrition may be limiting. We did find substantial differences between individuals and Banks in the nutrient contents of milk samples; these differences were largely overcome by pooling. These data demonstrate that infants fed DM that has not been pooled may experience dramatic differences from one day to the next in the composition of the milk they are fed—especially if the milk is from different individual donors. While the effects of nutrient variability are largely normalized in pooled PDM, there remain some deficiencies. This is especially important in the preterm population, because poor somatic growth correlates with low mental developmental scores and development of cerebral palsy [42].

Milk collected at milk banks in the United States is variable and is not a “single point in time”, but likely to have been collected longitudinally over several months. Each milk bank is pasteurizing different volumes of milk with varying mothers in a pool. For example, the OhioHealth Mothers’ Milk Bank typically pasteurizes 1500–2000 ounces per day from 4 to 10 different mothers, each having varying amounts of milk. The number of moms in a pool is dependent upon the available volume and calorie content of each. Current pooling practices utilizing HMBANA guidelines normalize the composition of PDM such that minimal differences in fatty acids or amino acids are observed among pooled samples and no correlations with lactational stage remain [36]. This observation may be explained by the fact that the sample size for pooled samples was one third that of the individual samples, and consequently there was less power to detect differences. Another explanation could be that the statistical differences between Banks Centers in the individual samples are largely driven by the outliers (up to five-fold different) and not actual differences from site to site. Despite the normalized contents in pooled samples, the absolute values vary two-to-three-fold and may still constitute a stressor on the infant. These findings would support customized supplementation for infants fed PDM to optimize nutrients.

DHA is a long chain poly-unsaturated omega-3 fatty acid that is a fundamental lipid in the human cortex and gray matter, and is correlated to developmental scores in preterm infants [43,44]. Furthermore, low concentrations of whole blood DHA have been correlated to late-onset sepsis and chronic lung disease, indicating the importance of DHA in overall infant health [45]. Throughout the last trimester of pregnancy, DHA is accreted at a rate of 50–75 mg/kg/day [46]. Infants born before the last trimester miss this accretion period and current DHA concentrations typically fed in the neonatal intensive care unit (NICU) fall short of the fetal accretion levels. Depending on diet, human milk concentrations of DHA can range from 0.2 to 2 mol % [28,47]. In the current study, measured levels of DHA in the regional PDM samples ranged between 2.22 and 20.20 mg/100 mL (equivalent to 0.08 and 0.67 mol %). This demonstrates that DHA levels in PDM are insufficient to consistently provide intrauterine accretion levels for preterm infants. In a previous study, supplementation of donors with 1 g of DHA per day resulted in human milk DHA concentrations that reached intrauterine accretion levels for the infant (0.8 mol %) [48]. These data suggest that mothers donating milk—especially milk to be used for preterm infants—should be advised to increase their daily intake of DHA, or direct supplementation of the PDM milk may be required.

Typical clinical practice is to increase total protein intake to improve growth without consideration of the concentrations of specific amino acids. Most requirements for amino acids are established for parenteral nutrition, and few address the needs for the enterally-fed infant [49]. Lysine is an essential amino acid used in protein synthesis and along with methionine is a precursor for carnitine synthesis, which is essential for fatty acid metabolism. After total protein, lysine is the amino acid most rate-limiting for growth and is essential to the biological value of food sources [49,50]. Huang et al. has demonstrated that infants require 130 mg/kg/day of lysine for optimal growth [50]. We observed substantial variability across Banks among amino acid contents in individual milk samples, but this variability was no longer evident in pooled milk samples. Several of the individual amino acids were lower than that recommended for parental nutrition (even in the pooled samples), but the relevance for enteral feeding is unknown (tyrosine, 74 mg/k/day; methionine, 49 mg/k/day; threonine, 33 mg/kg/day) [49]. Most importantly, lysine concentrations ranged from 52.1 to 66.3 mg/150 mL (the amount consumed by a healthy preterm per day), which is lower than the recommended 130 mg/kg/day assuming that most preterm infants weigh between 0.5 and 1 kg. Since many essential amino acids have defined functions, supplementing PDM with a specific quality of protein to ensure these amino acids are available may be as important as increasing total protein content.

One limitation of our study is that donated milk was from a wide range of lactational stages and included newborn milk to 12 months post-birth. Our study captured a median of 1 to 5.5 months, which can vary widely in protein and lipid content. A targeted donor process could perhaps improve the amino acid and fatty acid profile. The strength of our study is that it included a cross-sectional prospective examination of many regions in the United States to mimic current clinical practice to evaluate baseline nutrition in the donor milk.

## 5. Conclusions

The occurrence of failure to thrive currently witnessed in many infants fed PDM is concerning [18], and requires a further examination as to the adequacy of PDM as a sole source of nutrition. Our data indicate that individual PDM is highly variable, and that pooling lessens this variability; however, infants fed pooled PDM may still experience nutritional differences that may affect their overall growth. Our data supports the observed insufficiency of DHA and now lysine contents in most human milk across the United States and the need for supplementation—specifically in the case of the preterm infant. These findings support further studies on personalized maternal and infant supplementation strategies to meet the needs of high-risk infants receiving PDM [51].

## Figures and Tables

**Figure 2:**
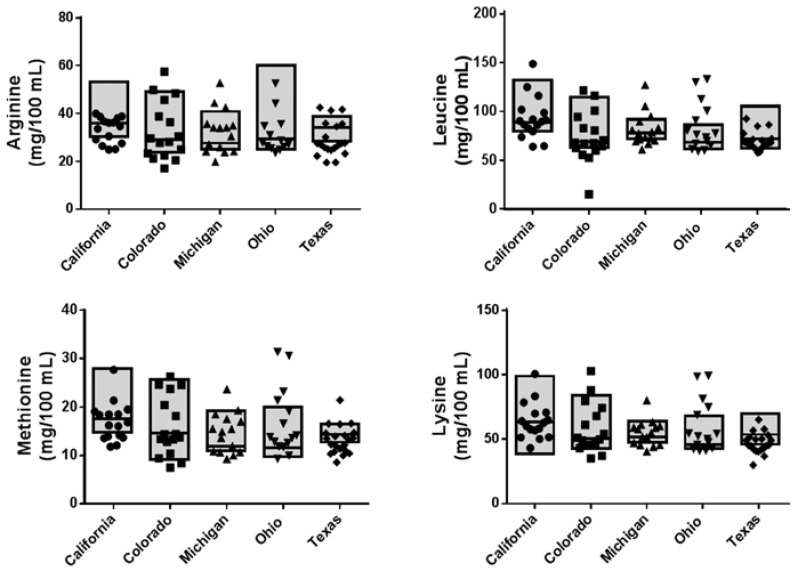
Amino acid levels in pasteurized donor milk samples. The individual variability of arginine, leucine, methionine, and lysine were calculated as mg/kg/day, assuming 150 mL per day and a 1 kg infant. Individuals within each Bank are represented by the small symbols. The boxes represent the median and range for the pooled samples. Data are representative of 15–16 individual samples and five pooled samples per Bank.

**Table 1:** Age and lactational stage of donors by milk bank.

Region	*n*	Age				Lactational Stage (months) *			
		Median	Range	Mean	Std. Dev.	Median	Range	Mean	Std. Dev.
California	16	33.5	24–41	32.9	5.3	4.0	1–13	4.8	3.4
Colorado	16	31.5	25–38	31.7	3.6	1.0 ^#^	1–8	2.2	1.9
Michigan	15	32.0	26–41	33.5	4.9	3.0	1–12	4.4	3.8
Ohio	15	30.0	20–42	31.7	5.9	2.0 ^#^	0–11	2.7	2.8
Texas-Ft Worth	16	32.0	25–37	31.2	4.4	5.5	2–11	6.1	2.7

* Data analyzed by Kruskal–Wallis, *p* = 0.002, with Dunn’s Multiple Comparison test; ^#^ different than Texas. Std. Dev. = standard deviation.

**Table 2:** Fatty acid levels in pooled samples obtained from Regional Milk Banks.

mg/100 mL	California		Colorado		Michigan		Ohio		Texas		*r*^2^ (Adjusted)	*p*
	mean ± SD	med.	mean ± SD	med.	mean ± SD	med.	mean ± SD	med.	mean ± SD	med.		
**C10:0**	19.0 ± 8.5	17.6	26.1 ± 10.8	23.7	22.2 ± 7.3	21.1	15.3 ± 5.0	12.2	28.9 ± 13.6	31.6	0.055	0.314
**C12:0**	118 ± 31.0	117	130 ± 60.0	108	134 ± 28.5	125	123 ± 35.1	116	151 ± 45.8	134	0.151	0.864
**C14:0**	144 ± 32.5	142	156 ± 63.8	135	182 ± 33.6	184	162 ± 43.6	147	172 ± 37.8	153	0.140	0..835
**C16:0**	553 ± 86.8	580	614 ± 159	577	666 ± 1125	651	570 ± 79.6	569	677 ± 179	601	0.005	0.456
**C16:1ω7**	68.4 ± 14.4	71.3	68.9 ± 20.3	59.3	76.1 ± 13.8	75.1	54.1 ± 8.21	58.3	74.0 ± 26.0	68.1	0.047	0.331
**C18:0**	184 ± 34.1	191	193 ± 36.8	178	214 ± 55.4	195	193 ± 32.0	197	239 ± 67.7	202	0.051	0.322
**C18:1ω9**	935 ± 189	983	923 ± 157	880	1019 ± 173	1004	1008 ± 216	975	1134 ± 275	1033	0.019	0.396
**C18:1ω7**	69.5 ± 15.8	63.3	81.9 ± 18.0	72.8	85.6 ± 20.9	75.4	74.1 ± 16.9	79.2	89.7 ± 35.7	81.3	0.050	0.581
**C18:2ω6**	494 ± 90	483	510 ± 57	506	593 ± 82	584	661 ± 171	626	603 ± 156	562	0.127	0.184
**C18:3ω6**	6.64 ± 0.68	6.49	6.63 ± 0.62	6.58	7.16 ± 0.82	7.13	4.42 ± 1.04	4.55	6.49 ± 2.45	5.73	0.241	0.065
**C18:3ω3**	44.2 ± 7.47	46.8	44.9 ± 3.26	45.3	48.5 ± 7.46	47.3	52.1 ± 12.8	51.7	57.8 ± 21.4	51.1	0.044	0.338
**C20:4ω6**	18.1 ± 4.97	18.1	14.9 ± 2.50	13.9	15.4 ± 1.41	15.1	16.3 ± 4.23	15.8	12.7 ± 1.56	12.7	0.074	0.274
**C20:5ω3**	3.74 ± 0.47	3.55	3.80 ± 0.53	3.53	3.15 ± 0.82	2.94	2.20 ± 0.44	2.35	3.56 ± 2.51	2.36	0.016	0.403
**C22:6ω3**	7.81 ± 1.62	7.82	7.29 ± 1.58	7.46	6.17 ± 2.00	5.62	8.20 ± 3.02	6.89	6.09 ± 2.63	5.48	0.066	0.627

Data were analyzed by multivariate regression with Bank as the fixed variable and lactational stage as a co-variate. Multivariate tests (Pillai’s Trace) revealed statistical differences between Banks (*p* = 0.000) across the model. Between-subject effects are indicated on the table. Data are representative of five pooled samples perBank; med = median. Data in gray indicates statistical significance at 0.05.

**Table 3:** Amino acid levels in pooled samples obtained from Regional Milk Banks.

mg/100 mL	California		Colorado		Michigan		Ohio		Texas		*r*^2^ (Adjusted)	*p*
	mean ± SD	med.	mean ± SD	med.	mean ± SD	med.	mean ± SD	med.	mean ± SD	med.		
**Phosphoserine**	97.8 ± 41.4	80.4	85.5 ± 28.0	84.4	80.2 ± 13.4	73.3	82.2 ± 23.1	81.1	83.1 ± 17.2	74.9	0.050	0.333
**Taurine**	8.44 ± 3.63	8.17	7.00 ± 4.72	8.06	4.89 ± 3.42	2.59	5.5 ± 2.6	9.00	8.3 ± 4.9	6.57	0.051	0.332
**Aspartic Acid**	102 ± 24.4	94.4	88.2 ± 26.1	82.6	83.6 ± 13.0	79.9	83.4 ± 8.8	74.6	87.5 ± 22.8	85.9	0.097	0.240
**Threonine**	44.1 ± 13.0	39.8	33.4 ± 10.0	29.3	35.9 ± 3.25	34.8	35.0 ± 6.58	34.0	34.4 ± 7.96	32.5	0.130	0.185
**Serine**	56.9 ± 14.4	52.7	46.2 ± 14.1	44.4	44.8 ± 8.21	41.1	44.8 ± 5.02	38.4	47.3 ± 14.4	43.5	0.105	0.227
**Glutamic Acid**	206 ± 43.4	185	173.8 ± 37.0	162	171 ± 14.2	164	179 ± 23.46	151	164 ± 23.7	175	0.152	0.156
**Proline**	101 ± 25.8	83.5	78.9 ± 25.2	66.2	81.4 ± 5.71	79.7	79.3 ± 20.1	67.1	66.6 ± 6.81	70.3	0.205	0.096
**Glycine**	25.2 ± 5.9	23.0	22.3 ± 5.85	20.5	21.3 ± 2.26	21.0	20.5 ± 3.5	20.0	23.4 ± 7.17	21.0	0.027	0.385
**Alanine**	47.1 ± 11.4	44.7	39.5 ± 13.1	37.9	38.1 ± 7.51	35.0	37.4 ± 3.87	31.6	40.3 ± 13.5	36.4	0.066	0.299
**Valine**	41.1 ± 12.6	36.9	30.9 ± 9.45	30.7	32.2 ± 4.32	33.0	27.2 ± 9.96	23.4	25.9 ± 7.56	28.1	0.140	0.172
**Methionine**	19.7 ± 5.8	17.6	15.1 ± 6.48	14.6	14.2 ± 3.86	11.9	14.4 ± 1.38	11.5	13.8 ± 4.59	14.3	0.212	0.090
**Isoleucine**	37.4 ± 12.7	32.9	27.0 ± 8.69	25.1	28.8 ± 3.08	27.7	28.4 ± 6.71	23.6	23.8 ± 3.35	25.5	0.199	0.102
**Leucine**	99.1 ± 24.0	88.1	76.8 ± 21.2	68.6	79.4 ± 7.85	77.5	76.5 ± 16.8	67.8	72.6 ± 10.8	71.2	0.221	0.082
**Tyrosine**	55.6 ± 20.9	52.1	44.1 ± 19.5	44.6	40.4 ± 11.6	33.1	44.6 ± 6.6	32.2	42.1 ± 17.0	45.1	0.006	0.469
**Phenylalanine**	38.1 ± 11.4	34.4	29.7 ± 8.90	26.5	30.0 ± 3.65	29.3	29.8 ± 5.10	25.1	30.4 ± 9.03	29.8	0.098	0.238
**Tryptophan**	188.7 ± 39.6	176	172 ± 28.6	169	173 ± 9.39	173	173 ± 29.8	161	157 ± 16.4	158	0.064	0.624
**Lysine**	66.3 ± 23.2	62.9	54.8 ± 16.7	50.1	54.0 ± 7.41	51.1	54.5 ± 8.8	45.3	52.1 ± 11.4	53.2	0.022	0.399
**Histidine**	22.4 ± 6.3	18.7	18.1 ± 4.78	16.4	53.2 ± 78.2	18.6	19.0 ± 4.14	16.1	17.5 ± 3.31	17.5	0.042	0.564
**Arginine**	38.7 ± 9.8	36.0	31.7 ± 10.3	29.2	31.3 ± 7.6	27.6	33.8 ± 3.85	29.3	37.3 ± 15.5	34.1	0.038	0.360

Data were analyzed by multivariate regression with Banks as the fixed variable and lactational stage as a co-variate. Multivariate tests (Pillai’s Trace) revealed no statistical differences between Banks or lactational stage across the model. Between-subject effects are indicated on the table. Data are representative of five pooled samples perBank; med = median. Data in gray indicates statistical significance at 0.05.

## Supplementary Materials

The following are available online at http://www.mdpi.com/2072-6643/9/3/302/s1, Table S1: Fatty acid levels in individual samples obtained from Regional Milk Banks. Table S2: Amino acid levels in individual samples obtained from Regional Milk Banks.
