# Supplementary material for: Lactational Stage of Pasteurized Human Donor Milk Contributes to Nutrient Limitations for Infants
Source: Nutrients. 2017 Mar 18;9(3):302. doi: 10.3390/nu9030302 (PMC5372965; doi:10.3390/nu9030302)
Supplement: Supplementary file 1 [file nutrients-09-00302-s001.pdf]

Supplementary Materials: Lactational Stage of Pasteurized Human Donor Milk Contributes to Nutrient Limitations for Infants

Christina J. Valentine, Georgia Morrow, Amanda Reisinger, Kelly A. Dingess, Ardythe L. Morrow and Lynette K. Rogers

Table S1. Fatty acid levels in individual samples obtained from Regional Milk Banks.

mg/100 mL	California		Colorado		Michigan		Ohio		Texas		r^2 *(adjusted)*	*p*
	mean ± SD	*med*	*mean ± SD*	*med*	*mean ± SD*	*med*	*mean ± SD*	*med*	*mean ± SD*	*med*		
**C10:0**	19.1 ± 9.8	16.8	25.1 ± 14.8	20.7	29.5 ± 11.5	24.4	23.6 ± 15.0	19.3	25.5 ± 13.9	23.2	0.000	0.424
**C12:0**	113 ± 52.0	96.0	113 ± 50.7	114	166 ± 60.9	143	158 ± 91.3	124	148 ± 65.8	139	0.014	0.302
**C14:0**	135 ± 56.0	118	137 ± 47.3	131	218 ± 78.6	207	200 ± 109	172	169 ± 59.0	169	0.063	0.074
**C16:0**	512 ± 210	474	548 ± 154	564	813 ± 181	811	681 ± 245	616	685 ± 221	616	0.134	0.006
**C16:1□7**	63.5 ± 26.5	62.0	69.6 ± 29.6	60.7	92.8 ± 23.0	94.3	64.2 ± 23.3	65.0	75.3 ± 22.9	70.6	0.135	0.006
**C18:0**	171 ± 73.9	159	190 ± 57.5	192	256 ± 70.4	252	220 ± 58.0	219	234 ± 78.9	206	0.114	0.013
**C18:1□9**	883 ± 323	832	913 ± 257	944	1241 ± 283	1233	1060 ± 277	969	1122 ± 301	1092	0.115	0.013
**C18:1□7**	67.7 ± 25.2	64.5	78.3 ± 24.5	82.1	104 ± 29.1	109.7	90.9 ± 25.9	73,1	88.1 ± 27.8	94.4	0.124	0.009
**C18:2□6**	469 ± 179	461	614 ± 134	541	753 ± 202	761	701 ± 189	665	663 ± 178	717	0.234	0.000
**C18:3□6**	6.56 ± 1.45	6.30	6.52 ± 1.81	6.30	8.32 ± 2.60	7.71	5.15 ± 3.00	4.98	6.46 ± 2.22	5.77	0.137	0.005
**C18:3□3**	88.8 ± 31,6	92.1	92.5 ± 14.5	96.1	123 ± 35.2	126	116 ± 30.1	106	106 ± 34.4	102	0.125	0.009
**C20:4□6**	30.1 ± 8.7	28.5	31.1 ± 8.3	31.2	37.5 ± 9.9	36.6	31.6 ± 9.5	32.1	32.8 ± 6.0	30.9	0.122	0.010
**C20:5□3**	3.76 ± 0.65	3.56	3.99 ± 1.15	3.51	3.28 ± 0.92	3.04	2.23 ± 0.74	2.28	2.77 ± 0.71	2.60	0.121	0.010
**C22:6□3**	7.22 ± 3.15	6.51	8.00 ± 5.09	5.82	7.24 ± 5.95	6.39	7.22 ± 3.82	5.88	8.02 ± 4.61	6.96	0.020	0.259

Data were analyzed by multivariate regression with Center as the fixed variable and lactational stage as a co-variate. Multivariate tests (Pillai's Trace) revealed statistical differences in Center ($p = 0.000$) and lactational stage ($p = 0.026$) across the model. Between-subject effects are indicated on the table. Data in gray indicates statistical significance at 0.05 (med = median).

Table S2. Amino acid **levels** in individual samples obtained from Regional Milk Banks.

mg/100 mL	California		Colorado		Michigan		Ohio		Texas		r^2 *(adjusted)*	*p*
	mean ± SD	*med*	*mean ± SD*	*med*	*mean ± SD*	*med*	*mean ± SD*	*med*	*mean ± SD*	*med*		
**Phosphoserine**	91.2 ± 32.2	78.8	95.9 ± 34.5	82.7	82.5 ± 20.8	80.5	70.2 ± 16.2	65.90	92.1 ± 36.1	79.2	0.247	0.000
**Taurine**	6.14 ± 3.12	5.78	7.60 ± 4.79	7.51	5.29 ± 3.50	3.60	4.83 ± 3.27	5.09	8.29 ± 5.86	7.21	0.061	0.079
**Aspartic Acid**	97.1 ± 21.3	95.4	93.6 ± 26.7	83.9	86.0 ± 15.9	87.3	75.2 ± 13.2	71.8	97.1 ± 39.3	78.5	0.194	0.001
**Threonine**	41.3 ± 10.5	39.6	36.0 ± 13.0	30.7	36.1 ± 5.38	35.8	31.8 ± 6.17	30.0	40.9 ± 14.6	35.5	0.180	0.001
**Serine**	52.5 ± 11.4	52.0	48.5 ± 15.4	43.0	45.4 ± 8.75	47.1	40.2 ± 7.40	38.7	53.8 ± 23.74	42.52	0.181	0.001
**Glutamic Acid**	198 ± 37.0	190	183 ± 43.6	171	172 ± 20.7	169.3	162 ± 21.2	158	180 ± 45.9	162.7	0.185	0.001
**Proline**	95.0 ± 23.7	89.9	83.3 ± 28.3	71.2	82.9 ± 12.6	82.6	73.3 ± 11.8	71.7	76.2 ± 20.1	70.1	0.139	0.005
**Glycine**	24.4 ± 5.8	24.2	24.1 ± 6.36	22.2	21.98 ± 3.48	21.3	18.6 ± 3.42	17.4	25.7 ± 11.2	20.0	0.198	0.006
**Alanine**	43.7 ± 9.00	44.7	41.8 ± 13.5	37.4	38.66 ± 7.96	40.1	33.3 ± 6.03	33.3	44.6 ± 20.9	34.5	0.157	0.003
**Valine**	36.9 ± 8.62	36.3	31.1 ± 13.9	27.4	31.6 ± 4.57	30.7	30.6 ± 9.00	26.3	40.8 ± 20.2	32.9	0.128	0.008
**Methionine**	17.0 ± 4.03	16.5	15.9 ± 6.20	14.1	14.6 ± 4.12	14.9	13.2 ± 2.93	13.2	16.8 ± 6.98	13.8	0.187	0.001
**Isoleucine**	34.0 ± 9.57	30.8	28.6 ± 12.5	23.9	28.6 ± 5.01	27.7	26.0 ± 5.96	24.5	28.6 ± 7.40	28.1	0.161	0.002
**Leucine**	93.3 ± 22.0	90.1	73.9 ± 26.0	68.0	81.6 ± 17.1	77.6	72.2 ± 13.9	68.0	83.9 ± 24.6	76.2	0.182	0.001
**Tyrosine**	48.4 ± 13.0	48.7	45.2 ± 16.3	41.2	41.9 ± 12.5	39.6	36.1 ± 8.73	35.2	47.0 ± 24.1	33.3	0.119	0.011
**Phenylalanine**	35.2 ± 8.40	35.0	30.0 ± 13.5	26.0	31.8 ± 8.64	31.3	26.6 ± 4.99	24.9	34.9 ± 14.1	28.8	0.139	0.005
**Tryptophan**	182 ± 39.9	171	172 ± 40.7	167	172 ± 22.0	167.1	169 ± 26.9	165	179 ± 35.3	184.3	0.051	0.108
**Lysine**	64.1 ± 14.3	61.5	58.2 ± 19.3	50.5	54.8 ± 9.99	55.0	48.3 ± 10.0	45.6	59.7 ± 20.1	52.3	0.198	0.000
**Histidine**	21.1 ± 5.52	19.6	19.0 ± 6.44	16.1	18.8 ± 3.02	18.8	17.0 ± 3.30	16.2	20.1 ± 6.34	18.4	0.156	0.003
**Arginine**	35.0 ± 9.10	35.4	32.7 ± 12.1	28.9	32.6 ± 8.95	34.0	29.0 ± 6.93	27.1	41.3 ± 20.8	31.0	0.147	0.004

Data were analyzed by multivariate regression with Center as the fixed variable and lactational stage as a co-variate. Multivariate tests (Pillai's Trace) revealed statistical differences between Centers ($p = 0.000$) and lactational stage ($p = 0.000$) across the model. Between-subject effects are indicated on the table. Data in gray indicates statistical significance at 0.05 (med = median).
